# After Malaria Is Controlled, What's Next?†

**DOI:** 10.4269/ajtmh.14-0056

**Published:** 2014-07-02

**Authors:** David H. Walker

**Affiliations:** Department of Pathology, University of Texas Medical Branch, Galveston, Texas

Because words mean everything, the vocabulary of the goal of a coordinated impact on a disease is important. Let us review the working definitions of three words: eradication, elimination, and control.

Eradication represents permanently breaking the link of transmission of an agent, such that it no longer exists in circulation anywhere in the world and is not a threat to reemerge by natural means.^1^,^2^ Eradication is the highest goal and currently even theoretically possible for only those infectious diseases for which humans are the reservoir. Falling short of a stated goal of eradication has a history of engendering the people's loss of confidence in public health, and thus, eradication should be undertaken only with a plan that is likely to succeed. Eradication has been accomplished only for smallpox in humans and rinderpest in ruminant animals. Current programs to eradicate dracunculiasis and polio have excellent chances of success. Measles is also a feasible and worthy target of eradication.

In contrast, elimination represents the establishment of a geographic area free of a formerly endemic disease. Elimination has been achieved for some infectious diseases in regions as large as continents, but there is always the threat of reintroduction.

Control can be defined as a reduction in the incidence of a disease to a defined target level. Although control sounds like a less impressive goal, it is not easy to accomplish and indeed, is a very significant achievement. Control of malaria would be such an achievement.^3^

Where do we stand today in regard to falciparum malaria? Its incidence has been reduced significantly by rapid diagnosis and treatment, insecticide-impregnated bednets, and vector control. Although a highly effective vaccine has yet to be developed, significant progress in vaccine research has been made. Even implementation of a vaccine that is only 30% effective would have a major impact on control and result in many lives saved.

I do not know what the ultimate outcome of the efforts to control and eliminate malaria will be. If malaria were to be eradicated, a large portion of our society's membership would have to find other scientific problems to address. A century ago, there was a medical specialty called syphilology. Reading medical records from my hospital from that long ago era revealed what a major public health problem tertiary syphilis was. The discovery of penicillin led to effective treatment of primary and secondary syphilis, the near disappearance of the late effects of syphilis, and in fact, the disappearance of the field of syphilology. It can happen. We would celebrate the removal of malaria from the field of tropical medicine. If this eradication occurs, what would we tackle next?

The major strength of the American Society of Tropical Medicine and Hygiene relates to infectious diseases that afflict populations that are poor and reside in regions with limited resources. Our members are leaders in the scientific and clinical studies of vector-borne viruses, bacteria, protozoa, and helminths and their diseases, enteric infections, and other tropical infectious diseases. These fields need more scientific and clinical investigative effort today and have room for more persons' contributions if funds were available for their support. To foreshadow my ultimate conclusion, we all need to develop collaborations that are interdisciplinary, share opportunities and resources, and maximize the breadth and impact of our strategies and efforts.

Indeed, we really know less about the causes of suffering and death in the tropics than many believe. Even vital statistics of birth and death are unrecorded in many areas of the world, much less the accurate causes of disease and death. Some diagnoses, such as malaria, dengue fever, and typhoid fever, are often ascribed to patients' illnesses without laboratory confirmation. Under the shadow of the umbrella of these diagnoses, other diseases are lurking. I have found significant incidences of spotted fever and typhus group rickettsioses and ehrlichiosis among series of diagnostic samples of patients suspected to have malaria, typhoid, and dengue in tropical geographic locations, where these rickettsial and ehrlichial diseases were previously not even considered by physicians to exist.^4^–^8^ Control of malaria or dengue would reveal the presence and magnitude of other currently hidden diseases and stimulate studies to identify the etiologic agents.

In southeastern Asia, intensive studies of undifferentiated febrile diseases have documented that the incidences of scrub typhus and rickettsioses, including murine typhus, leptospirosis, and Japanese encephalitis, are as high as the incidence of dengue and in some studies, greater than the incidence of typhoid fever.^9^ In these studies, a specific diagnosis was not established in more than one-half of the subjects enrolled, despite the tremendous efforts.^10^ Thus, not only would greater knowledge of the true diagnosis improve the outcome for patients with more accurately diagnosed treatable life-threatening diseases, such as scrub typhus, but also, the large pool of cases without a diagnosis established at all would serve as a likely source for discovery of novel emerging infectious diseases.

Just before I graduated from medical school, the war on infectious diseases was declared to be over and indeed, won. In 1992, more than two decades later, a period during which scores of newly discovered disease agents were identified, the concept of emerging infections was promulgated by a very prominent publication from the Institute of Medicine.^11^ Our society's members have played important roles in the discovery of many novel, previously unknown pathogens, such as the agents of several viral hemorrhagic fevers.^12^,^13^ However, this success has not been a strategic initiative, but rather, it has been more like firemen responding to the call of a house fire. I am certain that emerging infectious diseases will always continue to appear. We should be more proactive programmatically in pursuing research programs for competitive peer-reviewed funding for investigation of unusual undiagnosed syndromes and earlier discovery of the infectious agents. Indeed, we could probe more deeply into nature itself. Who would have predicted that bats would be a reservoir of agents such as the filoviruses Ebola and Marburg, the coronaviruses of Severe Acute Respiratory Syndrome (SARS) and Middle East Respiratory Syndrome (MERS), and a new genus containing Nipah and Hendra viruses?^14^–^16^

Human immunodeficiency virus–acquired immunodeficiency syndrome (HIV-AIDS) was once an unrecognized tropical syndrome with a small geographic footprint in Africa and a low incidence. It spread around the world before we identified its etiologic agent, even more years passed until successful treatment was developed, and we are still pursuing an effective vaccine. One can only dream of the potential effect of earlier identification of HIV, recognition of the nature of the threat, and possibly, even the early control of HIV-AIDS before it became a pandemic. Are we missing the opportunity now to counter future infectious plagues before they spread?

The concept of neglected tropical diseases has had the marvelous effect of shining the spotlight and research support particularly on helminthic parasites. All that this attention has accomplished is laudable, but the list of neglected tropical diseases from the National Institutes of Health omits such agents as *Orientia tsutsugamushi*, the cause of 1 million cases of scrub typhus annually (a treatable life-threatening disease that lacks appropriate point-of-care diagnostic assays, basic knowledge of mechanisms of immunity, and a vaccine).^17^ Disability adjusted years of life lost (DALYs) are an attempt to quantify the importance, severity, and impact of disease, but this approach does nothing for tropical diseases that are so neglected that DALYs have not been calculated.

What are next targets for tropical diseases research and implementation of measures to their control other than expansion of work on the important diseases that our membership is addressing now?

My choices would be just those topics that I have mentioned.

(1) Emerging tropical infectious diseases, including the discovery of new ones before they are widespread.
(2) Neglected tropical diseases, including other important diseases in addition to the major helminthic and protozoal infections that are now being emphasized.

We also must consider how our society should appropriately address non-infectious diseases that are a serious burden in populations with poverty and low resources, such as trauma (as occurs in traffic crashes) and maternal–neonatal disorders. It is clear that chronic diseases are also becoming more prevalent in the tropics, and cost-effective care for them is needed in resource-limited settings. I will not tackle these issues at this time, but we should make deliberate decisions on what our society could and should do beyond our current scope.

There are several fields that I strongly believe our society should embrace. I will address two of them.

First, bioengineering, which develops low-cost technology that is appropriate for the level of care and the training of caregivers in low-resource settings.^18^
Second, veterinary science, including the One Health approach to tropical diseases, for which there are common gaps in knowledge of both human and animal diseases and also, animal diseases that are important for human nutrition regarding food animals in the tropics.

There are symposia at our society's meetings in which bioengineers and veterinarians make valuable presentations. However, there is not a critical mass of individuals to address the issues that are relevant and offer rich opportunities for progress that could be a benefit to the bioengineers, veterinarians, and other members of our society. The potential for progress in addressing additional problems for collaboration among bioengineers, veterinarians, other basic scientists, and physicians at the American Society of Tropical Medicine and Hygiene is tremendous. It would be to the great advantage of our society if formal subgroups of Bioengineering for Tropical Diseases and Veterinary Tropical Medicine and One Health were established.

The currently established subgroups are of great value to us. However, our society could serve as a bigger tent for more companion subgroups of basic scientists and clinicians. These subgroups are, indeed, smaller societies integrated into the American Society of Tropical Medicine and Hygiene. We must not only maintain them but also, devise strategies to expand the strengths of the existing subgroups in parasitology, vector biology, arbovirology, clinical tropical diseases, and especially, our newest subgroup of global health. This last subgroup has tremendous potential to expand and organize the multifaceted nature of this burgeoning variety of interests, particularly among students with a strong emphasis on implementation science and all of the diverse interests of global health's constituents. Leaders among bioengineers and One Health-oriented veterinarians are urged to step forward and develop these interest areas within our society. Indeed, we should recruit bioengineers and veterinarians to join us.

Therefore, where do we in the field of tropical medicine stand right now?

The reality is that years of a flat National Institutes of Health budget, the Congressional sequestration, which is expected to deepen, and hypercompetitiveness for funding have reduced our resources to address tropical diseases. This annual meeting has fewer governmental participants owing to travel restrictions at federal agencies. How can we make progress in reducing the burden of suffering in populations with poverty and low resources under the constellation of challenges posed by this situation?

It is my opinion that we can move the frontier of fundamental knowledge ahead and effectively implement our knowledge and newly developed tools to actually alleviate disease. The main strategy in which I believe strongly is enhanced quality as well as quantity of collaborations. Each of our efforts remains largely focused on each of our own abiding interests, the goals that are the topic of our own work. Indeed, we have had success with this approach, or we would not be together here at the Annual Meeting of the American Society of Tropical Medicine and Hygiene. However, we have missed the chance to share resources to strive to multiple goals synergistically.

For example, many of the diseases that we investigate are acute undifferentiated febrile illnesses. Many of these studies focus on only one disease or one agent. Fewer than 10% of patients in the conducted study may suffer from the disease of interest. Clinical specimens are collected on all patients enrolled in the study, diagnostic testing identifies the subjects of interest to the focused investigators, and the hypotheses and analyses are investigated on the selected patients' samples and the samples of a control group that does not have the disease of interest. Within the study population may be patients with half a dozen other diseases of significant importance and interest to other scientists.

Those other scientists could evaluate the samples of patients with their disease of interest, identify patients with yet other diseases in serious need of study, and analyze the unused clinical samples to investigate them. Unfortunately, virologists, bacteriologists, and parasitologists rarely collaborate. We should collaborate. Shared resources and the most advanced technologic skills of the various investigators would potentially benefit each set of investigators. More progress would be made at a lower expense overall.

A strength of our society is its multifaceted membership. There are both outstanding basic laboratory-based scientists and clinical physicians with access to affected patients. There could be even greater collaborations among them engendered by more encounters and stronger interactions at this annual meeting. Our challenge is to arrange opportunities for these interactions.

Earlier, I mentioned the desire to see a larger subgroup of bioengineers as members of our society. These scientists have the ability to design and fabricate prototype low-cost point-of-care diagnostic devices for the diseases that clinicians and basic scientists need to study. Collaboration between the subject matter experts on the etiologic agent and the bioengineers could result in the development of low-cost diagnostic devices. The clinical physicians and subject matter experts could validate the effectiveness of the devices compared with the gold standard method and with informed consent, obtain clinical samples that could be used to answer questions about the pathogenesis of and immunity to the disease in studies performed by the basic scientists.

We can survive the current funding conditions by collaborating better to perform the highest-impact projects that would be prioritized by the National Institutes of Health study sections for funding, because they incorporate the greatest strengths of each investigator. We could sell potential commercial partners on the existence of markets for our diagnostic assays, vaccines, or novel therapeutics for travel medicine, reemerging infections owing to global climate change, such as some arboviral infections, and emerging diseases, such as the family of coronaviruses represented by SARS and MERS as well as others as yet undiscovered.

Two important components of our society that will be important for its future strength and higher-impact collaborative research are, first, our international members from tropical countries and second, our members who are still in training as students and post-doctoral fellows.

The decision of the council to dramatically reduce the annual dues for members who reside in low- and low–middle-income countries and make permanent the reduction in annual dues for trainees was done with the aim of encouraging both international members from tropical countries and trainees to make our society their permanent principal professional identity. Professional identification with the mission of our society, the warm collegiality of annual gatherings, the infusion of cutting edge new knowledge gained from the presentations, and the opportunity to meet personally the leaders in tropical medicine are self-evident advantages. What must become significant outcomes of these annual meetings are more newly established collaborations and newly identified opportunities for exciting productive research. These opportunities include identifying the next career step for trainees and new research partnerships of international members and American and European investigators.

We must strive to identify opportunities that do not necessarily bear the label tropical diseases but can be justified to use to study and develop tropical disease countermeasures, such as we have done for the tropical pathogens that are on the National Institutes of Health and Centers for Disease Control and Prevention priority list of biothreats. As Principal Investigator of the National Institute of Allergy and Infectious Diseases Western Regional Center of Excellence for Biodefense and Emerging Infectious Diseases Research, I have a program of research that has included tropical disease agents, such as arthropod-borne alphaviruses and flaviviruses, Crimean–Congo hemorrhagic fever virus, Ebola and Marburg filoviruses, Rift Valley fever virus, Nipah and Hendra viruses, Lassa and Junin arenaviruses, *Rickettsia prowazekii*, *Burkholderia pseudomallei*, SARS coronavirus, cryptosporidium, *Coxiella burnetii*, *Yersinia pestis*, and *Brucella melitensis.* These efforts have developed candidate vaccines for West Nile, chikungunya, eastern equine encephalitis, and brucellosis that have advanced through non-human primate testing and advanced a Rift Valley fever vaccine. Significant progress has also been made in developing low-cost point-of-care diagnostic devices.

Translational product-oriented research for countermeasures against biothreats has been challenged by such maxims as “It's no longer one bug, one drug.” The wish for a silver bullet demanded by the Department of Defense and the National Institutes of Health may be achieved some day, most likely by serendipity. However, the greatest impact on a disease is prevention. Other than sanitation, the best preventive tool is vaccination, which by nature of the immunizing antigens, is limited in range of disease coverage. I personally believe that vaccines and diagnostics are ripe for successful development. I have been through hard funding times before and intend now to persevere to find a productive pathway to obtain support for vaccine and diagnostics development.

What is next? It will be even better days for application of the even more powerful scientific methods that are being developed to the problems that we seek to solve. By collaborating intensely and wisely with one another, we can be members of the teams that achieve these goals, and team is the key concept.
